# Inhibitory activity of pentacyano(isoniazid)ferrate(II), IQG-607, against promastigotes and amastigotes forms of *Leishmania braziliensis*

**DOI:** 10.1371/journal.pone.0190294

**Published:** 2017-12-27

**Authors:** Camila F. Amorim, Luiza Galina, Natália B. Carvalho, Nathalia D. M. Sperotto, Kenia Pissinate, Pablo Machado, Maria M. Campos, Luiz A. Basso, Valnês S. Rodrigues-Junior, Edgar M. Carvalho, Diógenes Santiago Santos

**Affiliations:** 1 Serviço de Imunologia, Complexo Hospitalar Universitário Professor Edgard Santos, Universidade Federal da Bahia, Salvador, Bahia, Brazil; 2 Centro de Pesquisas em Biologia Molecular e Funcional, Pontifícia Universidade Católica do Rio Grande do Sul (PUCRS), Porto Alegre, Brazil; 3 Programa de Pós-Graduação em Biologia Celular e Molecular, PUCRS, Porto Alegre, Brazil; 4 Programa de Pós-Graduação em Medicina e Ciências da Saúde, PUCRS, Porto Alegre, Brazil; 5 Instituto de Toxicologia e Farmacologia, PUCRS, Porto Alegre, Brazil; 6 Instituto Nacional de Ciência e Tecnologia em Doenças Tropicais, Salvador, Bahia, Brazil; 7 Instituto de Pesquisa Gonçalo Muniz – Fundação Oswaldo Cruz, Salvador, Bahia, Brazil; Instituto Oswaldo Cruz, BRAZIL

## Abstract

*M*. *tuberculosis* and parasites of the genus Leishmania present the type II fatty acid biosynthesis system (FASII). The pentacyano(isoniazid)ferrate(II) compound, named IQG-607, inhibits the enzyme 2-trans-enoyl-ACP(CoA) reductase from *M*. *tuberculosis*, a key component in the FASII system. Here, we aimed to evaluate the inhibitory activity of IQG-607 against promastigote and amastigote forms of *Leishmania (Viannia) braziliensis* isolated from patients with different clinical forms of *L*. *braziliensis* infection, including cutaneous, mucosal and disseminated leishmaniasis. Importantly, IQG-607 inhibited the proliferation of three different isolates of *L*. *braziliensis* promastigotes associated with cutaneous, mucosal and disseminated leishmaniasis. The IC_50_ values for IQG-607 ranged from 32 to 75 μM, for these forms. Additionally, IQG-607 treatment decreased the proliferation of intracellular amastigotes in infected macrophages, after an analysis of the percentage of infected cells and the number of intracellular parasites/100 cells. IQG-607 reduced from 58% to 98% the proliferation of *L*. *braziliensis* from cutaneous, mucosal and disseminated strains. Moreover, IQG-607 was also evaluated regarding its potential toxic profile, by using different cell lines. Cell viability of the lineages Vero, HaCat and HepG2 was significantly reduced after incubation with concentrations of IQG-607 higher than 2 mM. Importantly, IQG-607, in a concentration of 1 mM, did not induce DNA damage in HepG2 cells, when compared to the untreated control group. Future studies will confirm the mechanism of action of IQG-607 against *L*. *braziliensis*.

## Introduction

The American tegumentary leishmaniasis (ATL) is considered one of the most important infections among all the neglected tropical diseases [1], with an extremely high morbidity rate [2, 3]. It is endemic in 98 countries and 5 continents, with approximately 1.5 million cases occurring per year [4]. The promastigote forms exist in the gut of a sand fly and infect the host through the inoculation of infected saliva into the skin, whereas the metacyclic promastigote forms of the parasite penetrate into the macrophages, being transformed in amastigote forms [5]. The *Leishmania (Viannia) braziliensis* is the main causal agent of ATL and is associated with distinct clinical forms of the disease, such as cutaneous leishmaniasis, disseminated cutaneous, and mucosal leishmaniasis [6–8]. Cutaneous leishmaniasis is characterized by a well-delimited ulcer with raised borders, predominantly in the inferior limbs [9]. Approximately 3% of the patients with cutaneous leishmaniasis develop mucosal disease, mostly affecting the nose. Mucosal disease is a severe form of leishmaniasis leading to nasal septal rupture and disaggregation of the structures of the face [10, 11]. Disseminated cutaneous leishmaniasis is defined by the presence of 10 or more acneiform, papular and ulcerated lesions in at least two non-continuous parts of the body [12, 13]. Pentavalent antimonials (meglumine antimoniate and sodium estibogluconate), amphotericin B (AMB) and miltefosine are the main drugs used for treatment of leishmaniasis, and the pentavalent antimony is the more common leishmanicidal drug used nowadays. Its parenteral use and associated adverse reactions decrease therapy compliance, and failure to antimony treatment is on rise in ATL [14–16]. AMB is the most effective drug for the treatment of leishmaniasis but its adverse reactions, mainly kidney injury, limit its use [14–16]. Liposomal preparates significantly reduce the toxicity of AMB, but the high cost of this formulation is a limiting factor for the extended use, especially because the disease affects poor people [14, 16]. The discovery of new effective drugs against leishmaniasis with low cost and reduced toxic profile is highly desirable [17, 18].

The pentacyano(isoniazid)ferrate(II) (IQG-607) is an organometallic compound analog to isoniazid, which contains a cyanoferrate(II) moiety bond to isoniazid [19, 20]. It was first designed to inhibit the proliferation of isoniazid-resistant *Mycobacterium tuberculosis* strains [20], inhibiting enzymes involved in the mycobacterial type II fatty acid synthase (FAS-II) system [21], then blocking the cell wall formation [22, 23]. Its efficacy against *M*. *tuberculosis* had been demonstrated *in vitro* and *in vivo* [23,24]. Herein, we tested the possible potential of IQG-607 against *L*. *braziliensis* strains, aiming at identifying new effective and safe strategies to combat leishmaniasis.

## Materials and methods

### Drugs

The pentacyano(isoniazid)ferrate(II) compound (IQG-607) (S1 Fig) was synthesized according to Oliveira et al., (2006) [19]. For all treatments, IQG-607 was dissolved in saline solution (0.9% NaCl) immediately prior to use. Amphotericin B (AMB; Sigma-Aldrich) was used as a positive control to inhibit *L*. *braziliensis* proliferation. Methyl methanesulfonate (MMS; Sigma-Aldrich) or dimethyl sulfoxide (DMSO, Sigma-Aldrich) were used as positive controls in toxicity assays.

### Culture of *L*. *braziliensis* strains

*L*. *braziliensis* LTCP 18483, LTCP 20195 and LTCP 19512 strains were isolated from lesions of patients (residents of the endemic area of Corte de Pedra, Bahia, Brazil) presenting cutaneous, mucosal and disseminated forms of leishmaniasis, respectively, before the treatment onset. These strains present different genomic sequences in the loci of the chromosome 25 that indicates a cause-effect relationship between the strain and the outcome of the infection [25]. The parasites obtained from the lesions were collected and transferred to tubes with biphasic medium (NNN) supplemented with 10% fetal bovine serum and Schneider medium (LGC Biotechnology, São Paulo, Brazil). After the expansion and proliferation of parasites presented in the lesion, they were collected and then maintained cryopreserved at promastigote stage, in Schneider medium with DMSO 10% and 1% penicillin streptomycin and glutamine (Gibco BRL, Grand Island, New York, USA), by the Immunology Service. At the time of the experiments, parasites were thawed, maintained in Schneider medium supplemented with 10% FBS and 1% of antibiotics and incubated at 27°C in a humidified atmosphere containing 5% CO_2_. This study was approved by the Ethical Committee of Faculdade de Medicina, Universidade Federal da Bahia and all patients who allowed to have lesions aspirated for isolation of *Leishmania braziliensis* signed an informed consent.

### Evaluation of inhibition of *L*. *braziliensis* promastigotes proliferation

Parasites (3x10^5^), at stationary (infective) phase, were added in a 96-well plate with “u” bottom for 3 days at 27°C in the presence of AMB 10 μM, IQG-607 (at different concentrations), or without treatment. After this period, a solution of 1 mg/mL of XTT with 0.06 mg/mL of PMS (Sigma-Aldrich) in PBS pH 7.0 was added in each well and incubated for additional 4 h at 27°C, until the absorbance was determined in a spectrophotometer, at 450 nm. As XTT is a colorimetric method, and IQG-607 acquires a brownish coloration in an aqueous medium, Schneider’s medium spiked with IQG-607 was considered as a blank well. The results presented in this study represent experiments from three independent tests performed in triplicates.

### Half maximal inhibitory concentration (IC_50_) value

To calculate IC_50_ of IQG-607 against *L*. *braziliensis*, 3x10^5^ parasites/well of LTCP 18483, LTCP 20195 and LTCP 19512 were cultivated and incubated for 3 days in Schneider’s medium with the drug, at 27°C. The tested concentrations started in 1 mM, followed by serial dilution until 2 μM, with all conditions made in triplicates. After the incubation period, the number of parasites were counted directly in optical microscopy. IC_50_ value was calculated on GraphPad Prism version 5 by using a non-linear regression curve.

### Isolation of PBMC and differentiation into macrophages

Heparinized peripheral venous blood obtained from six healthy subjects (non-resident in leishmaniasis endemic areas, not infected with other pathogens or in use of immunosuppressant drugs) was used in this experiment. Peripheral blood mononuclear cells (PBMC) were separated by density gradient with Ficoll-hypaque Plus (GE Life Sciences). PBMC from the interface were collected and washed 3 times with saline solution. The cells were suspended in RPMI 1640 medium with L-glutamine and 25 mM HEPES (Gibco) supplemented with 10% FBS and 0.5% gentamicin at 10 mg/mL (Sigma-Aldrich). PBMC were plated in Lab-tek plates (Thermo Scientific Nunc) at 4x10^6^ cells/mL and incubated in 37°C and CO_2_. After 2 h, the cells were washed with saline to remove non-adherent cells, leaving only monocytes in complete RPMI. Monocytes were then incubated in 37°C and 5% CO_2_ for 7 days to allow differentiation to macrophages. The six healthy subjects included in this study signed an informed consent term before blood sampling. This study was approved for the Ethical Committee of the Federal University of Bahia (612-907/2014).

### Macrophage infection by *L*. *braziliensis*, treatment with IQG-607 and evaluation of amastigote proliferation

For macrophage infection procedures, *L*. *braziliensis* strains were centrifuged, and added to the macrophage-containing wells in a proportion of five parasites for one macrophage in RPMI medium. Two h later, parasites that did not infect the cells were washed with saline and the macrophages were then incubated at 37°C and 5% CO_2_ with complete RPMI containing AMB or IQG-607, at different concentrations. Twenty four h later, the wells were gently washed twice with sterile NaCl 0.9% solution, and then the drugs (in RPMI medium) were re-added in the wells. After additional twenty four h (total 48 h of incubation), the slides from Lab-tek plates were stained with Giemsa (NewProv), and the percentage of cells infected with *L*. *braziliensis* as well as the number of parasites inside 100 cells were evaluated by optical microscopy.

### Cytotoxicity of IQG-607 in human macrophages derived from PBMC

Aiming to evaluate the possible cytotoxic effects of IQG-607 in human cells, uninfected macrophages derived from PBMC from 6 healthy subjects were plated at 4 x 10^6^ cells per well and incubated in 37°C and 5% CO_2_ in the presence of IQG-607 at different concentrations (43, 108, 215, and 430 μM). Twenty four h later, the wells were gently washed twice with sterile NaCl 0.9% solution, and then the drugs (in RPMI medium) were re-added in the wells. After additional twenty four hours, cell viability was assessed by Trypan blue exclusion assay.

### Cytotoxicity of IQG-607 in eukaryotic cell lineages

We also observed the cytotoxicity of IQG-607 against other cell lines. Human hepatocellular carcinoma (HepG2), monkey kidney epithelial cells (Vero), and human keratinocytes (HaCat) were obtained from Banco de Células do Rio de Janeiro, Brazil. HepG2, Vero and HaCat cells were cultured in DMEM (Dulbecco’s Modified Eagle Medium, Gibco) supplemented with 10% FBS (Gibco) and 1% penicillin-streptomycin (Sigma) and 1% fungizone (Sigma). Cells were maintained in tissue-culture flasks at 37°C in a humidified atmosphere containing 5% CO_2_.

Cellular viability after incubation with IQG-607 was evaluated by the neutral red uptake assay, which measures the capacity to incorporate the neutral red dye in the lysosome of viable cells [26]. HepG2, Vero, and HaCat cells were plated at 2 x 10^3^ cells per well in a 96-well culture microtiter plate and allowed to adhere for 24 h. Treatments with IQG-607 were added and incubated for 72 h. Cells were then washed with PBS, before the addition of 250 μl of neutral red dye solution (25 μg/mL, Sigma) prepared in serum-free medium. The plate was incubated for additional 3 h at 37°C and 5% CO_2_. After incubation, the cells were washed with PBS, followed by incubation with 100 μl of a desorb solution (CH_3_COOH:EtOH:H_2_O, 1:50:49) for 30 min and gentle shaking to extract neutral red from the viable cells. The absorbance was analyzed at 540 nm using a microtiter plate reader. Cell viability was expressed as a percentage considering the untreated control cells as 100% [27]. The results are expressed as mean ± SD or the mean of four independent experiments performed in triplicates. Selectivity indexes were calculated as IC_50_ eukaryotic cell lineages/IC_50_ parasite strains.

### Genotoxicity assay

The alkaline comet assay was performed to evaluate possible genotoxic effects of IQG-607 [28]. Briefly, HepG2 cells were plated in a 24-well culture microtiter plate (5 x 10^4^ cells per well) in DMEM and allowed to adher for 24 h prior treatment with IQG-607. Incubation with MMS at 100 μM was used as the positive control group [29]. Sixty μL of HepG2 cell suspension was mixed with 180 μL of low-melting point agarose (Invitrogen) and scattered on two pre-coated microscope slides with normal agarose (Invitrogen). Cells were then lysed in freshly prepared ice-cold solution (10 mM Tris, 2.5 M NaCl, 100 mM EDTA with 10% DMSO and 1% Triton X-100), for 24 h. In the dark, the slides were then incubated for 20 min at 4°C with fresh buffer (300 mM NaOH e 1 mM EDTA) to induce DNA unwinding and to expose the alkali-labile sites. The electrophoresis was conducted for 20 min at 25 V and 300 mA. After neutralization, the slides were fixed and silver stained. One hundred cells were scored according to the size and amount of DNA present in the tail. Individually, the cells were given an arbitrary value of 0 (undamaged) to 4 (maximally damage) [30].

### Statistical analysis

The statistical analysis was performed using GraphPad Prism 5 (San Diego, USA). Differences were considered significant at the 95% level of confidence, with a significant difference at *P*<0.05.

## Results

### IQG-607 inhibits the proliferation of *L*. *braziliensis* promastigote forms

**Fig 1:**
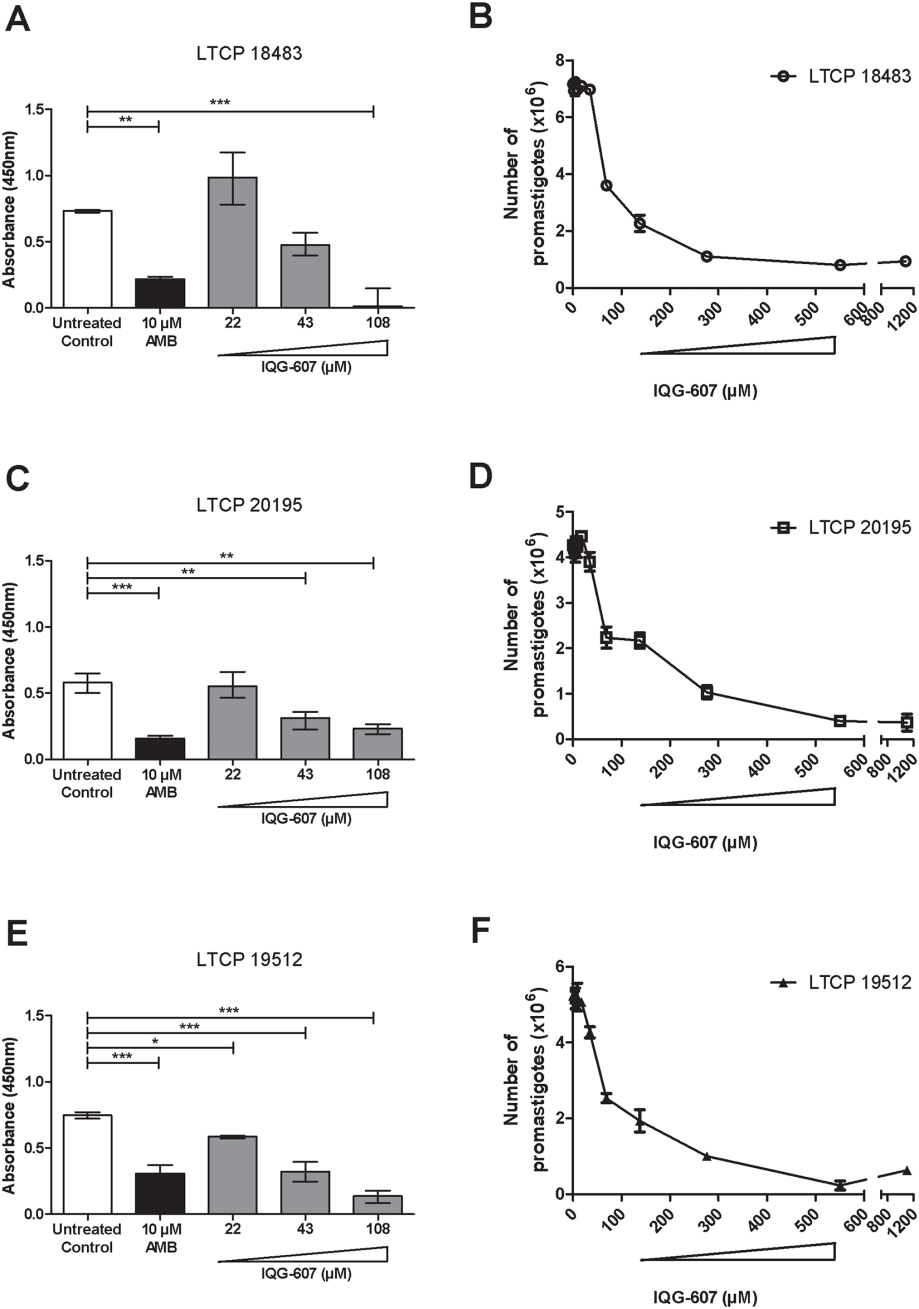
IQG-607 inhibits *L*. *braziliensis* promastigotes proliferation. Cell viability of *L*. *braziliensis* LTCP 18483 (A, B), LTCP 20195 (C, D) and LTCP 19512 (E, F) strains, isolated from lesions of patients presenting cutaneous, mucosal and disseminated forms of leishmaniasis, respectively, in the presence of AMB or IQG-607, after 72 h of incubation. Data is represented by mean±SD; One-way ANOVA followed by Bonferroni’s post-test were used in the statistical analyses, *P<0.05, **P<0.01 and ***P<0.001.

To evaluate whether IQG-607 was able to inhibit the growth of *L*. *braziliensis* promastigotes, the three different strains associated with cutaneous, mucosal and disseminated cutaneous of leishmaniasis were cultivated in the presence of the test drug. IQG-607 inhibited the proliferation of *L*. *braziliensis* promastigotes of all clinical forms of leishmaniasis in a similar way (Fig 1).

The treatment of *L*. *braziliensis* LTCP 18483 strain (cutaneous form) with AMB at 10 μM inhibited its growth by 71%, when compared to untreated control (*P*<0.01). Of note, IQG-607 treatment, at the concentration of 108 μM drastically decreased (99% of reduction) *L*. *braziliensis* viability (*P*<0.001) (Fig 1A). When evaluating LTCP 20195 (mucosal strain), the positive control AMB at 10 μM reduced 74% of its viability (*P*<0.001). Importantly, incubations with 43 and 108 μM of IQG-607 decreased 46 and 60% (*P*<0.01) parasite viability, respectively (Fig 1C). Regarding the LTCP 19512 strain, associated to a disseminated leishmaniasis, all concentrations of IQG-607 tested (22, 43, and 108 μM) resulted in statistically significant reductions (*P*<0.05 or *P*<0.001) in *L*. *braziliensis* viabilities (inhibitions ranging from 22 to 82%), while incubation of parasites with AMB 10 μM decreased 59% of cell viability (*P*<0.001) (Fig 1E). The IC_50_ values of IQG-607 ranged from 32 to 75 μM (Fig 1B, 1D and 1F). Comparatively, the concentration of 108 μM IQG-607 reduced the proliferation of promastigotes in 98% for LTCP 18483 cutaneous clinical form, 60% for LTCP 20195 associated with mucosal leishmaniasis and 82% for the LTCP 19512 isolated from disseminated cutaneous leishmaniasis patients.

### IQG-607 inhibits the proliferation of *L*. *braziliensis* amastigote forms

**Fig 2:**
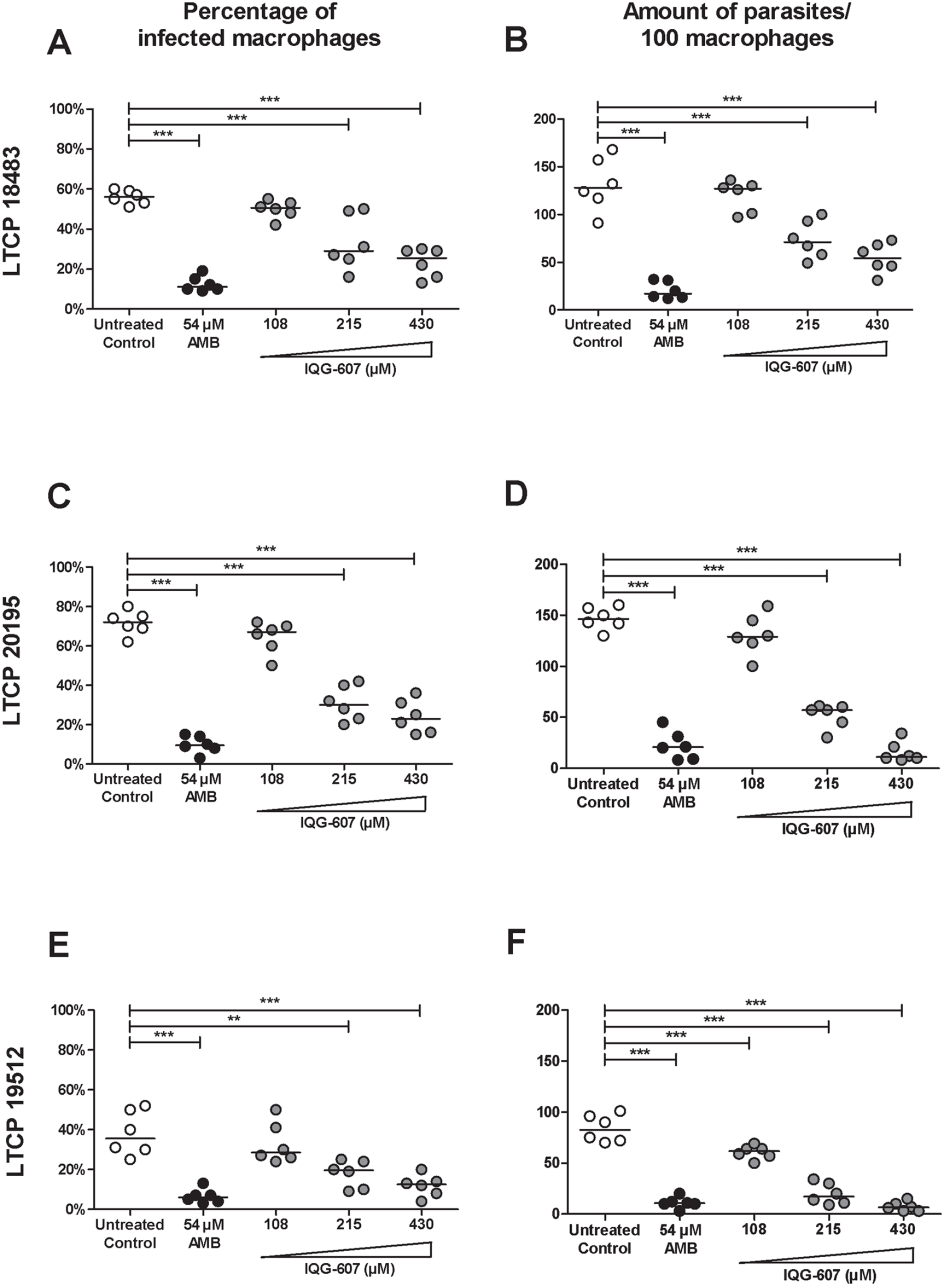
IQG-607 decreases the infection of macrophages by *L*. *braziliensis* amastigotes. Macrophages were infected with *L*. *braziliensis* (strains LTCP 18483 (A, B), LTCP 20195 (C, D) and LTCP 19512 (E, F) for 48 h in the presence or not of AMB and IQG-607. Percentage of infected macrophages and the number of parasites inside 100 cells are represented by median. One-way ANOVA followed by Bonferroni’s post-test were used in the statistical analyses, **P<0.01 and ***P<0.001.

Next, the influence of IQG-607 was evaluated on the proliferation of intracellular *L*. *braziliensis* from three different strains associated with cutaneous, mucosal and disseminated clinical forms of leishmaniasis. Prior infections, we determined the possible toxic effects of incubation with IQG-607 in PBMC, for 48 h. Regarding the ability of IQG to inhibit proliferation of amastigotes forms of *L*. *braziliensis*, macrophages from healthy individuals were infected with different isolates of *L*. *braziliensis* and treated with AMB or IQG-607. The percentage of infected macrophages and the number of parasites inside macrophages were determined (Fig 2). We observed that AMB at 54 μM decreased the percentage of infected macrophages with the LTPC 18483 strain from 56 to 11% (*P*<0.001). IQG-607 treatment at 215 and 430 μM decreased 29 and 25% of infected macrophages, respectively, and these reductions were statistically significant (*P*<0.001) (Fig 2A). Concerning the absolute numbers of amastigote forms inside the macrophages, the untreated control resulted in a median of 132 amastigotes/100 cells, while treatment with AMB (at 54 μM) reduced to a median of 17 amastigotes/100 cells (*P*<0.001). Amastigote burden medians from IQG-607-treated groups were 67 and 54 amastigotes/100 cells (treated with the concentrations of 215 and 430 μM, respectively), which were statistically significant decreases (*P*<0.001 for these two concentrations) when compared to the untreated group (Fig 2B).

Importantly, IQG-607 was also able to inhibit *L*. *braziliensis* amastigotes from the mucosal strain. Incubation with IQG-607 at concentrations of 215 and 430 μM decreased the percentages of infected macrophages from 72 to 28 (*P*<0.001) and to 23% (*P*<0.001), respectively, when compared to untreated group (Fig 2C). Regarding the number of amastigotes inside the macrophages, a median of 146 amastigotes/100 cells were counted in the untreated control, while treatments with IQG-607 at 215 and 430 μM significantly reduced the median burdens to 60 (*P*<0.001) and 8 amastigotes/100 cells (*P*<0.001) (Fig 2D).

For the disseminated strain, our results showed that AMB (at 54 μM) decreased the percentage of infected macrophages from 35 to 7% (*P*<0.001). IQG-607, at 215 and 430 μM, significantly reduced the mean percentages to 19 (*P*<0.01) and to 12% (*P*<0.001), respectively (Fig 2E). Moreover, amastigote burdens inside the macrophages were reduced from 84 (in the untreated control) to 61 (*P*<0.001), 20 (*P*<0.001) and to 7 amastigotes/100 cells (*P*<0.001), after incubation with 108, 215 and 430 μM, respectively (Fig 2F). Comparatively, the concentration of 430 μM, for 48 h, reduced in 58% the number of amastigotes of the strain for LTCP 18483, 95% for LTCP 20195, and 91% for LTCP 19512.

### Investigation of possible cytotoxic effects of IQG-607

**Fig 3:**
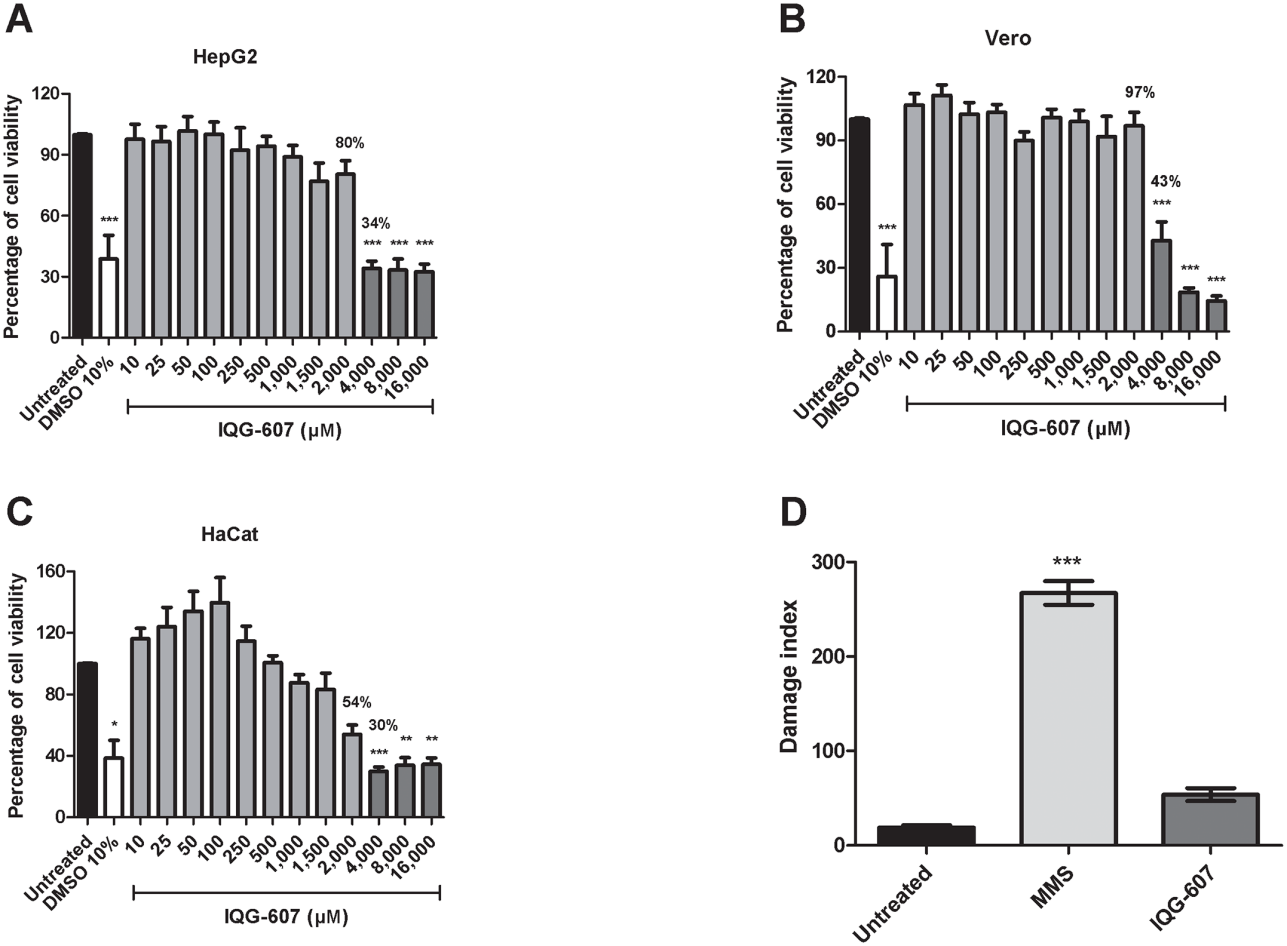
Cytotoxic activity of IQG-607 against cell lines. HepG2 (A), Vero (B) e HaCat (C) cells were treated with IQG-607 for 72 h and cell viability was assessed by neutral red uptake assay. DNA damage index (D) were measured by alkaline comet assay in HepG2 cell after 24 h of IQG-607 (1 mM) or MMS (0.1 mM; positive control) exposure. Data is represented by mean±SD. One-way ANOVA followed by Bonferroni’s post-test were used in the statistical analyses, *P<0.05, **P<0.01 and ***P<0.001.

When evaluating a new drug candidate, it is mandatory to investigate the toxicological properties and safety profiles of the new molecule. We tested the cytotoxicity of IQG-607 against macrophages derived from PBMC. After 48 h, the percentage of viable cells was accessed by Trypan Blue reagent assay. Incubations with IQG-607, from 43 to 215 μM, did not affect cell viability (S2 Fig). Moreover, we investigated the cell viability of three additional different lineages, Vero, HaCat and HepG2 cells, after incubation with IQG-607, for 72 h. Of note, concentrations of IQG-607 from 10 to 2,000 μM did not significantly affect the cell viability, according to assessment of the three lineages. Treatment with DMSO at the concentration of 10% was used as positive control, and induced cell death by 60–70%. Treatments with the concentrations of 4, 8 or 16 mM reduced cell viability to percentages lower than 50%, for the three lineages tested (Fig 3A–3C). We then estimate that the IC_50_ is higher than 2 mM, for the eukaryotic cell lines tested. The selectivity indexes are higher than 25 when we consider the values of IC_50_ (ranging from 32 to 75 μM) for the parasite strains (promastigote forms). Moreover, the selectivity index is estimated to be close to 10 when we analyzed the results from the amastigote assays. In our experiments, the concentration of IQG-607 of 215 μM significantly reduced the percentage of infected macrophages, and reduced the amount of parasites (in 100 macrophages) for the three strains of *L*. *braziliensis* tested.

Additionally, we aimed at investigating the genotoxic potential of IQG-607 by the alkaline comet assay. Interestingly, IQG-607, even when tested in a high concentration (1 mM), did not induce DNA damage in HepG2 cells, when compared to the untreated control group (Fig 3D). MMS, a known mutagenic alkylating agent and was used as positive control for DNA damage.

## Discussion

Different species of Leishmania are able to cause ATL. Depending on the host and the parasite virulence factors, the disease may present as full healing small cutaneous ulcers, with or without an involvement of nasal, pharynx, and larynx mucosa or by the presence of multiples lesions (upon 1000 cutaneous lesions), as observed in disseminated cutaneous leishmaniasis. Therapy for leishmaniasis is a challenge in drug discovery, since a very few drugs have been shown effective in controlling the disease. However, most of treatments are administered by parental route, making their use unfeasible in poor endemic rural areas in South America [14–16]. Moreover, most of available drugs for leishmaniasis are associated to relevant adverse effects. Herein, we showed that IQG-607 is an affective agent against promastigote and amastigote forms of *Leishmania braziliensis*, and could be used as starting point for the development of a new drug for leishmaniasis.

Enzymes involved in fatty acid and sterol metabolism have been considered to be attractive pharmaceutical targets in *Leishmania*, as well in other members of the kinetoplastida group [31]. In leishmaniasis, the genome project identified some candidate genes for type II fat acid synthase enzymes such as β-ketoacyl-ACP reductase, β-ketoacyl-ACP synthase and enoyl-ACP reductase, that could mediate fatty acid synthesis in these parasites [32–34]. In the present study, we evaluated *in vitro*, the ability of IQG-607, a known inhibitor of enoyl-ACP reductase enzyme from *M*. *tuberculosis* [19, 20], to influence in Leishmania survival. We used 3 isolates that were associated with 3 distinct forms of the disease, since *L*. *braziliensis* is polymorphic and we have previously shown that these genotypic differences are due to changes in the loci of the chromosome 25 [25]. It is known that promastigotes are more sensitivity to leishmanicidal drug than the amastigotes, and we initially evaluated the effects of IQG-607 in this form of the parasite. IQG-607 was effective in significantly reducing the proliferation of the 3 isolates at the concentration of 108 μM. Similar to the observations with promastigote forms, IQG-607 was able to decrease Leishmania proliferation of the 3 isolates inside macrophages.

*In vitro* sensitivity tests of *L*. *braziliensis* to drugs were recently standardized [35, 36]. The polymorphism of Leishmania from the same species and its importance in different clinical presentations of the disease has been recently documented for *L*. *braziliensis* [25]. However, no previous study has evaluated the sensitivity of the isolates from cutaneous, mucosal and disseminated patients to new drug candidates. The response to therapy of these three clinical forms is quite different. While the failure rate to antimony therapy of cutaneous leishmaniasis ranges from 25 to 75% [37], 50% of the mucosal leishmaniasis patients fail to cure with one series of antimony [38] and there are few studies evaluating alternative treatments in disseminated cutaneous leishmaniasis. Moreover, failure in the therapy after use of AMB or miltefosine for mucosal leishmaniasis is high [38–40]. Based on previous findings we expected that the ability of IQG-607 to reduce Leishmania proliferation in isolates of mucosal and disseminated cutaneous patients was lower than that observed with cutaneous leishmaniasis isolates. Surprisingly, in this study we report that the effect of IQG-607 seems to be higher in isolates of mucosal and disseminated leishmaniasis. Furthermore, toxicity assays revealed a very favorable profile for IQG-607, according to assessment of cell lineages or primary culture cells. This is in accord with previous publications showing a very good safety profile for IQG-607 in mice, rats, and minipigs [41–43]. Noteworthy, IQG-607 did not exhibit genotoxic effects, as revealed by the alkaline comet assay, further reinforcing the favorable toxicological profile of this molecule.

The limitation of this study was the use of only one isolate of *L*. *braziliensis* from each form of the disease. Our data clearly show that IQG-607 is effective in inhibiting proliferation of promastigotes and amastigotes forms of *L*. *braziliensis*. IQG-607 could be considered a potential safe drug for treatment of *L*. *braziliensis* infection and future work will elucidate the mechanism of action of IQG-607 against Leishmania.

## Supporting information

S1 Fig Chemical structure of pentacyano(isoniazid)ferrate(II) (IQG-607). The pentacyano(isoniazid)ferrate(II) complex, [FeII(CN)^5^(inh)]^3-^, an octahedral complex containing isoniazid bound to an pentacyanoferrate(II) center through its pyridinic nitrogen.
(TIF)

S2 Fig Cytotoxic activity of IQG-607 against macrophages derived from PBMC. PBMC were cultivated in the presence of (A) AMB or (B) IQG-607 at different concentrations to evaluate the cytotoxic potential of this drug. Data represent the median of the percentage of dead cells after 48 h of incubation. One-way ANOVA followed by Bonferroni’s post-test were used in the statistical analyses, **P<0.01.
(TIF)
