# Supplementary figures and images for: Inhibitory activity of pentacyano(isoniazid)ferrate(II), IQG-607, against promastigotes and amastigotes forms of Leishmania braziliensis
Source: PLoS One. 2017 Dec 27;12(12):e0190294. doi: 10.1371/journal.pone.0190294 (PMC5745003; doi:10.1371/journal.pone.0190294)

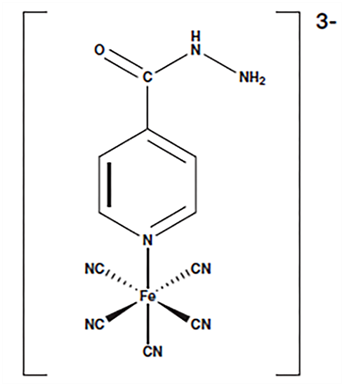

Supplement: S1 Fig — The pentacyano(isoniazid)ferrate(II) complex, [FeII(CN)5(inh)]3-, an octahedral complex containing isoniazid bound to an pentacyanoferrate(II) center through its pyridinic nitrogen. (TIF) [file pone.0190294.s001.tif]

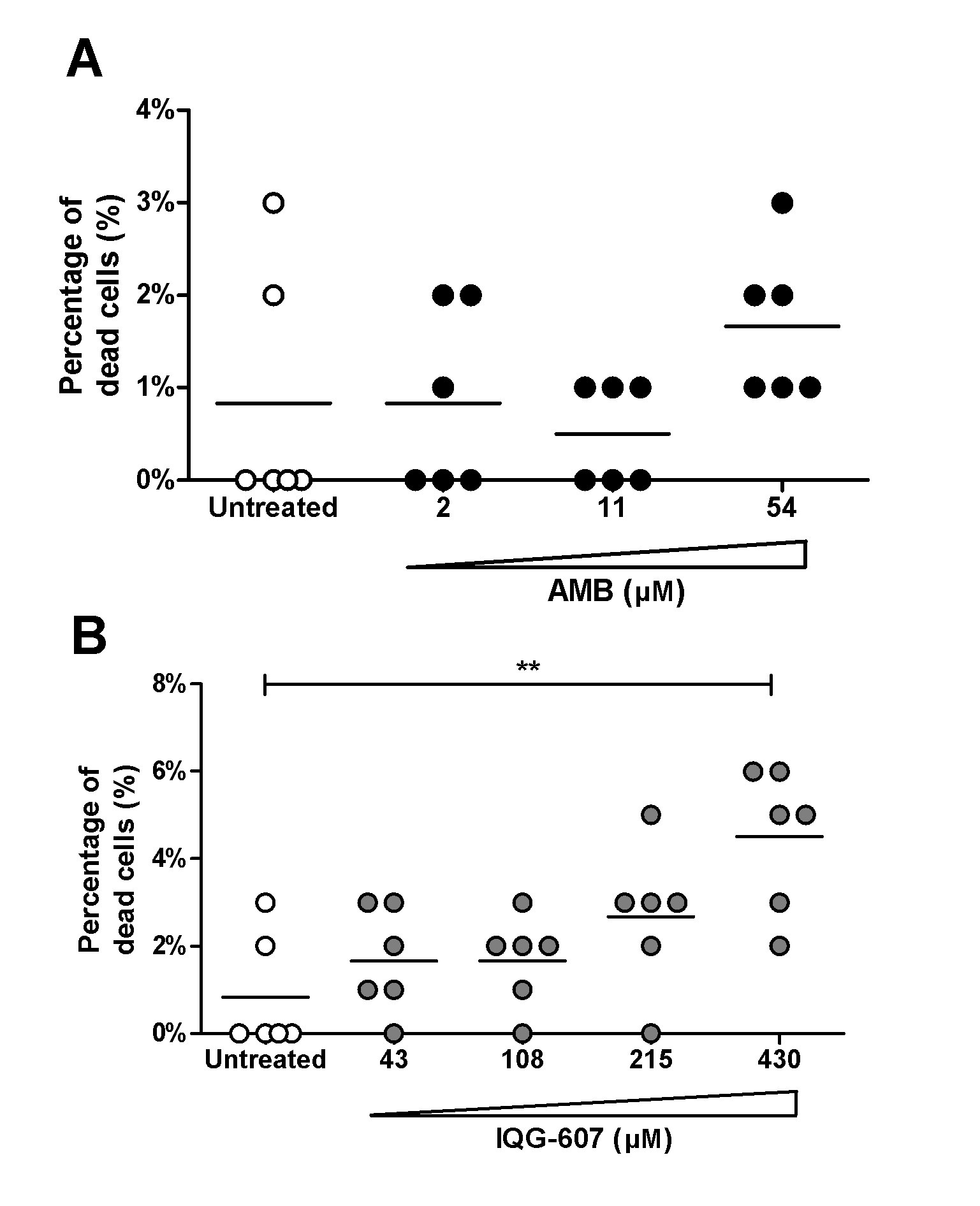

Supplement: S2 Fig — PBMC were cultivated in the presence of (A) AMB or (B) IQG-607 at different concentrations to evaluate the cytotoxic potential of this drug. Data represent the median of the percentage of dead cells after 48 h of incubation. One-way ANOVA followed by Bonferroni’s post-test were used in the statistical analyses, **P<0.01. (TIF) [file pone.0190294.s002.tif]
